# Communication Matters—Pitfalls and Promise of Hightech Communication Devices in Palliative Care of Severely Physically Disabled Patients With Amyotrophic Lateral Sclerosis

**DOI:** 10.3389/fneur.2018.00603

**Published:** 2018-07-27

**Authors:** Katharina Linse, Elisa Aust, Markus Joos, Andreas Hermann

**Affiliations:** ^1^Department of Neurology, Technische Universität Dresden, Dresden, Germany; ^2^German Center for Neurodegenerative Diseases (DZNE) Dresden, Dresden, Germany; ^3^Interactive Minds Dresden GmbH, Dresden, Germany

**Keywords:** amyotrophic lateral sclerosis, augmentative and alternative communication technologies, eyetracking, brain-computer-interfaces, quality of life, end-of-life-decisions

## Abstract

Amyotrophic lateral sclerosis (ALS) is the most common motor neuron disease, leading to progressive paralysis, dysarthria, dysphagia, and respiratory disabilities. Therapy is mostly focused on palliative interventions. During the course of the disease, verbal as well as nonverbal communicative abilities become more and more impaired. In this light, communication has been argued to be “the essence of human life” and crucial for patients' quality of life. High-tech augmentative and alternative communication (HT-AAC) technologies such as eyetracking based computer devices and brain-computer-interfaces provide the possibility to maintain caregiver-independent communication and environmental control even in the advanced disease state of ALS. Thus, they enable patients to preserve social participation and to independently communicate end-of-life-decisions. In accordance with these functions of HT-AAC, their use is reported to strengthen self-determination, increase patients' quality of life and reduce caregiver burden. Therefore, HT-AAC should be considered as standard of (palliative) care for people with ALS. On the other hand, the supply with individually tailored HT-AAC technologies is limited by external and patient-inherent variables. This review aims to provide an overview of the possibilities and limitations of HT-AAC technologies and discuss their role in the palliative care for patients with ALS.

## Introduction

Amyotrophic Lateral Sclerosis (ALS) is the most common motor neuron disease. It is characterized by progressive degeneration of upper and lower motor neurons, leading to progressive paralysis, dysarthria, dysphagia and increasing respiratory disabilities. The average survival after diagnosis is 3–5 years and most common causes of death are respiratory failure or dysphagia. Therefore, life-prolonging measures and especially tracheostomy might significantly increase survival (1, 2). As there is still no curative therapy available the main focus is palliative care aiming to improve ALS-patients' individual quality of life (QoL) and support caregivers (3). Moreover, it is reported that multidisciplinary integrated palliative care not only improves QoL but even prolongs survival (4, 5). On the one hand, physical symptoms such as pain, increasing swallowing and respiratory difficulties and restrictions in activities of daily living can at least be partially controlled by medication and support for everyday life, e.g., by the use of assistive devices. Potentially life-sustaining measures such as percutaneous endoscopic gastrostomy (PEG), non-invasive ventilation (NIV) and tracheostomy with invasive ventilation (TIV) can also improve QoL by controlling feeding problems or dyspnea. However, their initiation needs careful discussion, individualized decisions and patients' explicit and fully informed consent (6–9).

Data is sparse for other countries than Japan, but TIV-rates among ALS-patients seem to increase up to 20% (10, 11). These numbers underpin the relevance of the complex and extremely difficult decision whether or not—and if yes, at which physical or psychological health status—life-prolonging measures should be terminated. The few studies on this subject describe that patients often decide to terminate TIV because of a subjective “loss of meaning in life” and poor QoL (12, 13). At best, this issue should be discussed and considered explicitly in patients' advance care planning after careful discussion (6).

On the other hand and in face of the fatal and progressive nature of the disease, palliative care for people with ALS needs to address not only control of somatic symptoms but also psychological, spiritual and existential aspects. Decision-making over medical care from the time of diagnosis until death is a cyclic process that should be guided by patients' autonomy and care has to be adapted to the changing needs of patients and their families. To meet these needs, intense communication between the affected persons and health professionals is essential (14–16). Communication is further described as crucial to sustain hope and reduce fear in palliative care (17).

Overall, dysarthria occurs in 80–95% of people with ALS at some point in their disease course, making them unable to meet their daily communication needs by means of natural speech (18, 19). We thus aim to provide an in-depth overview of the possibilities of HT-AAC technologies and their influence on, patient care, social life and QoL of severely disabled patients and caregivers, but also of their limitations. On this basis, we discuss the role HT-AAC use in palliative care for patients with ALS.

## Importance of high-tech augmentative and alternative communication (HT-AAC) technologies in ALS

In line with Janice Light's description of communication as “the essence of human life” (20), a qualitative study of McKelvey et al. (21) impressively described the frustration and sadness that patients and their partners experience as speech deteriorates: “That was probably the biggest hurt. She couldn't talk.” Patients are often deprived of their ability to judge, experience a lack of control and a change of their social roles. The ability to communicate is strongly associated with patients QoL (22) and communication is seen as crucial for the adaption to terminal diseases such as ALS (23). Therefore, while verbal as well as nonverbal communication abilities deteriorate, augmentative and alternative communication (AAC) strategies and technologies become more and more important. AAC strategies in general are in place to support communication related to a large variety of issues, such as personal and medical care, social interaction and closeness, community involvement and employment, and to express personality and feelings (18, 21).

AAC might be no- or low-tech (gestures, facial expressions, handwriting, topic boards, alphabet boards, and eye-linking partner-supported systems) or high-tech with or without synthesized speech output (e.g., tablets, touchpads, head- or limb-movement-activated microswitch systems). High-tech augmentative and alternative communication (HT-AAC) technologies afford minimal or no head or limb movement and enable complex, caregiver-independent communication as motor abilities decrease (24, 25). Since the decision for TIV increases survival and therefore the length of HT-AAC-use (26) and considering the growing percentage of patients deciding for TIV, the need for HT-AAC will likely grow. The use of (HT-)AAC devices to support communication in different groups of severely disabled patients has been discussed since decades [e.g., (27)]. This review focuses on HT-AAC for severely disabled patients with ALS who depend on multimodal palliative care.

## Technology of HT-AAC eyetracking computer systems

The most promising and best-studied HT-AAC devices are eyetracking computer systems (ETCS) which allow cursor control by eye movement. Eye movements are often the least fatiguing (28, 29) if not the only remaining volitional movements that allow communication in ALS (27).

Although the technology that drives eye operated speech generating systems has been modified over the course of the last 40 years, the underlying principles did not change much. All systems use infrared sensitive cameras, mostly based on complementary metal-oxide-semiconductor sensors nowadays and with an active infrared light source to illuminate the eyes. The systems primarily differ in the relative positioning of the infrared light source with respect to the camera lens axis. Systems with an infrared light source located very close to the camera lens axis make use of the bright pupil effect: the infrared light gets reflected on the eye's retina and produces a bright image of the pupil. Conversely, in systems with the infrared lights placed off axis the images received from the camera sensor generate dark pupils. In both systems the infrared light source produces additionally a bright reflection on the cornea (the glint), which is together with the pupil center used to calculate the pupil-glint vector (30, 31). This vector then serves to calculate the Point of Regard on a computer screen and thus can be used to type by means of a gaze sensitive on-screen keyboard or to drive other computer functions.

## Advantages and promises of HT-AAC in palliative care

### Acceptance and usability of HT-AAC to restore communication ability

Several studies demonstrate the positive impact of ETCS-use for severely disabled people. First of all, acceptance and user satisfaction are reported to be high in ALS and traumatic brain injury (32, 33). Ball et al. (32) found in a study of 50 patients with ALS that 96% of those who were recommended AAC technology due to increasing communication disabilities accepted the device, either immediately or after some delay. The three main reasons for their decision for AAC were maintenance of communication, participation in community and employment. Patients who rejected AAC often suffered from frontotemporal dementia (FTD), which is in line with another study (18).

Patients use ETCS for a variety of activities such as face-to-face-communication—even in groups—e-mail contact, internet access and other computer functions and programs as well as for environment control (24, 34, 35). Regarding the increasing importance of social media, their access by HT-AAC is an additional valuable mean of communication, link to the outside world and thereby supports patients' social networks (36). Thus, HT-AAC can enable social and intellectual stimulation, independent leisure activities and the patients to express even complex thoughts. As AAC allow even severely disabled patients to communicate with less familiar caregivers, they enrich patients' possibilities in choosing communication partners.

Evaluation studies of HT-AAC-use show that once a functioning ETCS could be established, patients use it for several hours each day and report a high user satisfaction, preservation of communicative abilities and subjective indispensability of the device (34, 37). Interestingly, the worse patients' clinical conditions, the higher seems to be their acceptance of HT-AAC (38).

### Regain of social participation, psychological wellbeing and quality of life

In an interview study by McKelvey et al. (21) spouses reported that AAC technologies helped to maintain the emotional connection within families. What they additionally valued as a very precious function was that patients—with the help of their next of kin—could discuss philosophical ideas and author “last words” and thoughts to their families. The use of AAC devices even enables psychotherapy for severely disabled patients in order to reduce psychological distress and promote autonomy and self-esteem (39).

Several cross-sectional and two longitudinal studies found a positive association between higher psychosocial wellbeing or QoL and the use of ETCS (25, 34, 38, 40–42). The assumption of a positive effect on Qol is further supported by the findings that HT-AAC-use serves as an active coping mechanism, helps patients to express emotions and personality and to maintain social roles, participation in family and community and even employment (21, 43). These results are confirmed by the first study evaluating the association between QoL and ETCS use in ALS-patients in the locked-in-state (LIS) in a fully caregiver-independent manner by using ETCS-based assessment (44). Patients reported on average a high QoL and the study suggests that ETCS preserve patient autonomy and therefore psychosocial wellbeing particularly by enabling social activities, which patients named as the most important area of life for their QoL. A generally high subjective QoL in ALS has been reported before for less severely affected patients as well as for LIS-patients (45–47). It is discussed that psychological wellbeing might even modify disease course in ALS (48, 49).

Consequently it can be assumed that by enabling patients to stay mentally autonomous and realize their needs in terms of social activities and participation, encouraging successful adaption to the disease and thereby increase psychological wellbeing, HT-AAC might even have disease-modifying effects. This remains to be clarified in longitudinal investigations.

### Reduced caregiver burden

Caregivers of ALS-patients report low QoL and high burden (50–53), which is especially true for tracheotomized patients and those in LIS (8, 44). The use of ETCS though is associated with reduced caregiver burden, probably by improving patient autonomy and making patient-caregiver-communication more effective (40). An interview-study of 34 family caregivers of ALS-patients reports a very positive attitude toward HT-AAC devices, an increased perception of social closeness and fewer difficulties in providing care due to the AAC-use. These benefits are greater for those with higher AAC technology skill levels (35). Corallo et al. (42) could demonstrate in a longitudinal study of 15 LIS-patients and their caregivers that enabling patients to communicate via HT-AAC reduced caregivers' anxiety and increased their vitality as well as social activity and social role functioning; results that highlight the positive value of HT-AAC supply also for the caregiver themselves.

### (Neuro)Psychological assessment

Another important issue is the use of HT-AAC for neuropsychological assessment, since neuropsychological test procedures usually require at least some motor or verbal skills and therefore become invalid for severely disabled patients. It is known that cognitive deficits affect a great proportion of all-ALS-patients (54) and can compromise their ability to judge and decide over their medical care and life-prolonging measures (see chapter Cognitive and behavioral impairment and its consequences for HT-AAC-use). Promising attempts have been made to develop ETCS-based test procedures of cognitive functioning (55, 56).

## Impact of patient autonomy in palliative care

The reported findings make clear that enabling complex communication independent of a “translation” by caregivers/next-of-kin and thus patient autonomy is crucial for the preservation of psychological wellbeing of severely disabled patients. Furthermore, HT-AAC have high implications for end-of-life-issues: First, the possibility to communicate might directly change patients' attitude toward life-prolonging measures, as Fager and colleagues explicitly reported for one LIS-patient equipped with a computerized communication system controlled by minimal head movements: “He was so encouraged [by the regain of his communicative abilities] that, when he entered the hospital with pneumonia, he changed his medical code status from ‘do not resuscitate’ to ‘full code’” (57). In turn, we assume for two of the patients who were recruited for our study (44) but died before the assessment that an earlier supply with ETCS could have changed their decision against life-prolonging measures.

Second, caregiver-independent communication enabled through HT-AAC supply is crucial for assessing the patients' psychological condition and actual will and ensure self-determination of care. Advanced care planning and in general decisions over the medical care for severely disabled patients need a careful discussion of all relevant procedures, advantages and potential risks to ensure self-determination. This is specifically true for decisions to initiate or terminate life-prolonging measures such as PEG, NIV or TIV. It was mentioned above that the patients' self-rated QoL is often relatively high, moreover and importantly it is significantly underestimated by caregivers as well as the general population (52, 58, 59). This is in particular noteworthy for patients in LIS: the average QoL of the 11 LIS-patients in our study, self-rated via ETCS, was 81% while next of kin estimated patients' QoL to be only 63% and thus similarly low as their own self-rated QoL of 54% (44). It is not clarified yet which factors contribute in which extend to this significant discrepancy. However, there has been reported a “response shift” in the evaluation of their QoL by patients toward a higher value of social activities and a lower value of financial aspects, mobility and leisure activities (47, 60, 61); a shift that patients' next of kin are apparently not aware of (44), maybe because it does not happen for them.

Furthermore, in the face of their shorter lifetime some patients gain a “deeper view” and a higher appreciation of life (62).

However, it must also be considered at this point that some patients may suffer a loss of awareness or insight in their situation or a reduced ability to judge it, as executive functioning (63–65) and social cognition including empathy are impaired in a proportion of non-demented patients (66–68); an issue that is further discussed in chapter Cognitive and behavioral impairment and its consequences for HT-AAC-use.

Irrespective of its causes, the contradictory assessment of patients' QoL by patients themselves and caregivers may have tremendous consequences on end-of-life-decisions and thus makes it essential to enable patients to communicate even complex utterances independently of their next-of kin or caregivers. Actually, LIS-ALS-patients themselves confirm that they are able to do so by means of their own ETCS but not without the device (37, 44). It is indispensable that patients' wishes concerning life-prolonging measures are not undermined. This is extremely difficult to assess and ensure, as communication structures in families and between health professionals and patients are hard to grasp anyway, all the more if one partner suffers from severe communication difficulties. We observed one case, in which the patients' wish for TIV probably was circumvented on hospital admission which caused her death (44).

Since the patients' will may change during disease course (12, 69, 70), communication must be enabled at every time point in the progression of the disease and thus even if no head or limb-movement or natural speech is possible. This is emphasized by the fact that a significant proportion or even clear majority of ALS-patients is tracheotomized unplanned, e.g., as an emergency measure, and in a relevant amount of cases without explicit informed consent of the patient (8, 71). As this can obviously be avoided by early, careful and detailed advanced care planning as recommended by Oliver et al. (15), the valid assessment of patients' will has to be striven for at each point of time. It was argued before that this approach will also disburden caregivers from vital decisions for their loved ones in the fear of making them against their actual will. Parallel to ensuring the patient's autonomy, the highly burdened caregivers need to be involved in medical decision making (6, 15) and to receive specialized practical and psychological support (72, 73).

## Limitations and pitfalls of HT-AAC-use and-supply

Nakayama et al. (74) suggested a definition of five stages of communicative abilities of TIV-ALS-patients that is of high value for the prediction of impaired communication: patients who can communicate without any high-tech devices are classified as stage I, patients with communication difficulties that can be overcome by use of HT-AAC technologies to a varying extent as stages II to IV and those who cannot communicate at all as stage V. Predictors identified for the progression from stage I to a higher one and therewith predictors of severely impaired communicative abilities are oculomotor dysfunctions, TIV and full quadriplegia.

This model indicates that despite the diverse possibilities and promising research results by far not all patients suffering from advanced ALS and other conditions that affect communication abilities are supplied with an HT-AAC device or respectively gain a successful restoration of their ability to communicate by means of HT-AAC. Beside the three important reasons for this lack identified by Nakayama et al. (74), there are several more which can be assigned to the three main components of the AAC-acceptance model by Lasker and Bedrosian (75): factors of the user, the environment and the device.

### Factors of the user

#### Eye pathologies and eye movement dysfunctions

A number of ophthalmologic diseases and oculomotor dysfunctions can complicate ETCS-use. Although oculomotor function is typically spared from the effects of ALS, dysfunctions occur in a proportion of the patients and particularly ophthalmoparesis in those with prolonged survival (74, 76). Certain deficiencies like slowed down saccades or ptosis can be accommodated by some ETCS, but others like eye movement paralysis as well as further problems such as glaucoma, gaze tiredness or problems to keep the head still can make it difficult or even impossible to use ETCS (34).

While normal astigmatism can be usually very well compensated during the calibration process, more severe and irregular deformations of the cornea may pose a challenge for accurately determining the user's gaze, since this may affect the way the infrared is reflected from the eye. If only one eye is affected, some ETCS allow focusing only on the eye without cataract. Nystagmus, a condition characterized by repetitive, uncontrolled eye movements, is another factor that can make it impossible to use ETCS because nystagmus (a) can impede calibration as a user is not able to hold its gaze still for a prolonged time and (b) even if calibration is possible, users will have difficulties resting their gaze on a button for a long enough period of time. Another condition that can interfere with ETCS use is strabismus. While single eye strabismus can as well be compensated by focusing only on the non-affected eye and eventually applying an eye patch on the other one, alternating strabismus cannot be compensated for by ETCS as it is not possible to determine which eye is directed at any given point in time.

Another frequent obstacle in clinical practice are spurious reflections from glasses since they may, depending on the location of the reflections, interfere with the corneal reflection. Although contact lenses are usually no problem, for hard contact lenses the corneal reflection sometimes happens to lye partly on the circumference of the lense and thus only partly on the cornea.

Electrooculogram-based eye-computer interfaces might overcome a few of the limitations of ETCS since they are not influenced by lighting or the physical conditions of the eyes. However, this method also requires the users' abilities to control their eye-muscles and is moreover less precise than ETCS (77). Microswitch-activated systems that rely on any residual muscular activity can be another option (78).

Irrespective of which HT-AAC device is chosen, there is still the risk that patients progress to a total locked-in-state (TLIS) and thus to stage IV of communication abilities (74), since TLIS is defined as the complete loss of muscle control including the eye muscles and therefore any valid ability to communicate needs (79). This is obviously an extremely burdening situation for caregivers and health professionals. The overall prevalence of TLIS is difficult to determine, but Hayashi and Oppenheimer (80) reported a prevalence in ALS-patients on TIV of 11.4%.

#### Psychosocial factors

Certain attitudes and needs are potential reasons for the refusal of HT-AAC by patients. In her qualitative study on the non-acceptance of HT-AAC Murphy (81) reported on this matter that some did not use their device because they desired using their own voice as long as possible. Communicating via a device was perceived as just “not the same.” In line with another case study, patients preferred the higher social closeness and the direct interaction of face-to-face-communication (81, 82). Furthermore, patients reported a “shared understanding” in everyday communication with familiar partners that makes HT-AAC devices dispensable. However, referring to the stage model of Nakayama et al. (74), all of these patients were still in stage I, so still able to communicate via speech which often changes as the disease progresses (81). Low-tech and face-to-face-communication—if (still) possible—might be more effective and comfortable for communicating quick needs and for interacting with familiar partners, while sharing detailed information and communication with less familiar partners requires HT-AAC (75, 83). In summary, advantages of different communication modes depend on individual abilities, aims of communication and familiarity of interlocutors.

An additional difficulty is the optimal timing of AAC-interventions, thus the decision at which stage of communication ability or impairment HT-AAC devices are introduced and established. On the one hand, patients and caregivers often don't want to be confronted with predictable deficits before speech becomes intelligible and thus delay the decision about HT-AAC use (18, 84). On the other hand, timely referral not only ensures punctual delivery of the device but also better learning conditions for the patient (14, 18).

Age, education and computer experiences might also influence HT-AAC acceptance. Actually, samples of the reported investigations on ETCS-acceptance and impact on wellbeing (25, 40, 44) were relatively young and highly educated compared to average ALS-patients. However, Caligari et al. (25) found no influence of education and computer experience on ETCS acceptance or benefit. Considering age as a potential factor, Spataro et al. (34) reported regular users to have a younger age of disease onset compared to irregular and non-users.

Cognitive deficits are another important influence factor on the usability of ETCS. While the progression rate of cognitive deficits to a full blown dementia in late stage ALS is not known, up to 10% of ALS patients suffer from FTD at any specific time (54) and cases of the development of severe dementia under TIV are known. Apart from that, studies describe cognitive deficits to be relatively stable over the disease course and observed good cognitive functioning in patients with late-stage ALS (85, 86). Nevertheless, mild to moderate cognitive impairment is highly prevalent in ALS, which is described in depth in chapter Cognitive and behavioral impairment and its consequences for HT-AAC-use.

### Factors of the environment

#### Supply and professional support of HT-AAC-use

The environmental conditions are probably the most vulnerable aspect of HT-AAC-provisioning for severely disabled people, regarding to begin with the supply of the devices and the continuous individual support to ensure their optimal usability. First, clinicians involved in the care need to be aware of HT-AAC devices and their possibilities and—concerning the mentioned issue of timing of supply—must support the patients' decision process on the use of such devices in an active but also sensitive and properly timed manner. This can be considered a difficult (84, 87) and important challenge, especially in view of the finding that lacking referrals by physicians are a frequent reason for delayed supply with HT-AAC devices (84).

Second, funding and availability of devices can be an issue as the health care system of many countries do not or only partially finance (HT-)AAC devices. The national health system in the United States started reimbursing AAC in 2001, but application is an exhausting and time consuming process (18). As Donegan et al. (88) report, the national health service of Italy started providing ETCS for ALS-patients several years ago because of increasing awareness brought by the research, but this is not consistent practice. For Germany, Funke et al. (89) found as a result of a cohort study on a case management program for ALS-patients that only 61% of AAC devices procured by the treating neurologists were finally delivered to the patients, which might be in fact an overestimation for the general population since the study was conducted in specialized ALS centers. The main cause of failed provision with a HT-AAC device was rejection by the health insurance, followed by rejection by the patient and patient's death. The mean latency of provision was 93 days, a long period of time for people not able to communicate without the device. The authors speculated that especially decisions over expensive assistive devices are guided by financial considerations at the expense of patients' wellbeing (89).

Moreover, provision of HT-AAC devices is not only costly but also difficult to install as they need to be adapted to each individual user. Service providers need to provide training of circa 5 h (24) and ongoing support, trouble-shooting and individual customizing over an extended time period (18, 33). Insufficient training is often reported to be a reason for helplessness and non-use of HT-AAC (33, 75, 81). Caregivers need detailed step-by-step-instructions and intense training too, because they serve as indispensable HT-AAC facilitators (90). AAC success is reported to depend on caregivers concerns, attitudes and awareness (81, 91) and caregivers with higher skills report higher reward (35).

To avoid unequal service provision and optimize the timing of AAC-interventions, regular assessments of patients' communication abilities by trained and independent AAC-experts are recommended (84). At best, an assistive technology clinic as described by Casey (92) is created, combining expertise, time and material resources and the ability to test and individually customize devices. This might also offer a solution for the challenge of optimal timing of AAC-interventions, by allowing patients to get familiar with different technologies and to face upcoming communication problems step by step (14). It is recommended that the communicative abilities of patients suffering from diseases leading to foreseeable disabilities are regularly evaluated by trained health professionals such as speech language pathologists (93). Patients should be referred for AAC assessment when their speaking rate falls below 100 to 125 words per minute or when patient or listener perceive the communication effectiveness as decreasing (18, 94).

#### Influence of family caregivers or next of kin

An issue that has not been addressed systematically in the literature until now is that caregivers might experience negative aspects of patients' HT-AAC use. The ability to communicate detailed thoughts and wishes, also with third parties, might lead to increased feelings of burden–especially in combination with a decision pro NIV. This was possibly the case for one patient in a study by Linse et al. (44), in which the family returned the ETCS without stating reasons and despite it was working well and the patient expressed the wish to use it. Evaluating the perceived usefulness of ETCS, next of kin also reported some critical issues, e.g., an increased burden since patients started to use the ETCS (37). Reasons for this higher burden need to be clarified. It is conceivable that it is related to patients' increasing duration and severity of ALS, to the social-communicative or technical requirements raised by the ETCS itself or to the fact that the patient is now able to communicate his wishes that she or he want to be satisfied by the next of kin.

However, family caregivers of severely disabled patients' are in general a highly burdened and overloaded population that has to be considered and supported in the palliative care (15) and in particular concerning patients' supply with HT-AAC. Beside and in connection with the discussed low quality of life and wellbeing of next of kin, it is known that severe diseases like ALS have far-reaching effects on a social system beyond the index patient; and it is therefore essential to study impacts on the caregivers and their perspectives separately from patients' perspectives (89). While HT-AAC technologies can help to prevent the patients' social networks, assuming the role of the caregiver often results in a loss of freedom and of time and energy for self-care as well as in a change of life plans. The size, quality and changes of their personal social network have to be investigated in future to minimize the negative consequences of the disease on family caregivers (95, 96) and in consequence to counteract unfavorable influences on patient's decision, e.g. for or against use of HT-AAC devices.

### Factors of the device

There are also several issues related to the HT-AAC device itself that can hinder its optimal use. Particularly for ETCS, accuracy of older devices can be insufficient and complex calibration and setup procedure can complicate the handling (24). The bad quality of the voice output is another issue occasionally regarded as problematic by patients as well as caregivers (21, 43, 81). Voice banking and voice conversion techniques lead to hope for more personalized speech synthesis in the future (97). However, we are not aware of a single study investigating the value of this voice banking technique. From own experience it can be reported that patients themselves experienced their recorded voice not as their own. In contrast, next of kin do so but have difficulties accepting that this technology device talks with the voice of the patient. Finally, independent of voice banking and concerning the authenticity of the voice, the speech output does not adapt to the content of the words in terms of emotion, thus e.g., joy and crying do not sound differently.

Another technical drawback of the currently used eyetracking technology, independent from individual factors of the patient (e.g., oculomotor dysfunction), is the sensitivity of the infrared light sensitive camera to ambient infrared light, because it immensely reduces the usefulness of the devices in outdoor settings. Only reliable and portable devices that can be adjusted e.g., to a variety of lightning conditions can ensure the use of HT-AAC in different settings (40, 43, 81). Another relevant difficulty in ETCS-use is the “Midas touch problem.” It describes the frequent phenomena that the focus of attention is not in accordance with the users' direction of gaze, which results in non-intended commands like for example a wrong selection of letters (98).

Ideally, switching of access methods (e.g., from touch to joystick to eyegaze) with one device and one easy-to-learn “intuitive” software as well as the setup of different individually tailored features (e.g., internet and mobile phone access, environment control, leisure activities) should be possible with one HT-AAC device. These options would allow to adjust the device to the patients' changing needs and physical abilities and enable communication with different partners in different settings (18, 91). High quality products should be employed as technical problems and learning difficulties reduce the motivation to use HT-AAC even though it is generally wished and needed (38).

## Cognitive and behavioral impairment and its consequences for HT-AAC-use

As suggested earlier, relevant behavioral and cognitive impairment under the threshold of (frontotemporal) dementia but also caused by frontotemporal dysfunction is a common and critical feature of ALS (99, 100). Guided by FTD-diagnosis though, there is a distinction between (non-dement) ALS with behavioral impairment (ALSbi), with cognitive impairment (ALSci) and with a combination of both (ALScbi) (99).

Cognitive impairment in general is reported to affect between 30 and 40% of the ALS-population (54, 64, 101), although estimations of prevalence vary considerably; an inconsistency that is probably partly explained by the considerable heterogeneity of first those deficits (63, 99) and second of the methods used for their assessment (65).

Nevertheless, impairment is consistently reported for the broad cognitive domains of executive functions, language and memory (54, 63, 64, 102, 103). Recent meta-analyses additionally confirmed deficits in social cognition as another prominent feature of ALS (54, 67). Furthermore, different behavioral changes can be observed in ALS-patients (104).

Concerning consequences for HT-AAC-use, Beukelman et al. (18) interestingly reported for patients with mild cognitive deficits that all who wanted and needed AAC for communication were able to use it. Anyway, in view of the cognitive, linguistic and social demands of communication, the cognitive and behavioral impairments due to ALS must be assumed to have important implications not only for communication ability in general (105) but also by (HT-AAC)-use in particular. This is most obviously for deficits in language comprehension and expression.

### Language impairment

Language function is a very broad domain, but Beeldman et al. (54) analyzed that studies reporting its impairment in ALS often operationalized it as the ability to name objects in Visual Naming Tests, which are used as an important diagnostic tool for aphasia (106, 107). Naming deficits probably based on a general impairment of basal word finding processes seem to be a typical aspect of language dysfunction in ALS (108). The capability to communicate effectively and comprehensible by means of HT-AAC can be further critically aggravated by a lack of comprehension and thus errors concerning semantic, syntax or grammar of language. Such problems were found to affect almost 50% of all ALS-patients (109), already in early disease stages (110) and even when executive functioning is intact (110, 111). They with single word and in particular verb processing (109, 112) and also with continuous speech production in form of e.g., less produced words, shorter utterances, and incomplete sentences (110, 111).

A function especially often reported to be strongly impaired in ALS is (phonemic and semantic) verbal (letter and category) fluency (54, 109, 113). Deficits of fluency in comparison to healthy controls are even present when performance is controlled for patients' reduced motor speed (65, 114). Such deficits can indicate a limited access or principal limitation of the mental vocabulary (115) or a broad semantic deficit (108) and therefore a serious restriction of communication ability.

Although these language function impairments were determined in spoken or written/typed language, they should as well compromise language production by means of HT-AAC devices in terms of comprehensibility, effectiveness, completeness, subjective meaningfulness and value for the recipient. Patients' deficits of language or speech comprehension should hamper communication anyway, irrespective of the means they use for it.

### Executive dysfunctions

Impairments of language function in ALS are reported to be strongly associated with executive dysfunctions (64, 109), some experts even construed them as a pure consequence of the latter (114). Executive function is the most extensively researched cognitive domain in ALS (109) and a population-based study and a meta-analysis confirmed highly prevalent deficits for a variety of standard neuropsychological tests in non-dement ALS-patients (63, 64). A significant lower performance compared to healthy subjects was also found for a complex measure of executive functioning with high ecological validity, controlled for patients' reduced motor speed (65). Generally spoken, executive functions are a group of higher cognitive functions with a crucial role for controlling basal cognitive functions (116) like attention and memory. Hereby, they are necessary for sorted and goal-directed behavior (117) in situations when automated, intuitive or routine behavior is not possible or inadequate (118) and assumed to be of great importance for response initiation and motivation (108). They are therefore obviously important for social interaction and communication (via HT-AAC).

Specific executive functions that are repeatedly reported to be impaired are shifting (114, 119–121) and working memory (114, 122–124), while patients show deficits for explicit measures of inhibition control in some investigations (124) but not in others (121). The high prevalence of verbal fluency deficits is mentioned above, but important again at this point. This is because tasks of verbal fluency and shifting are considered as measures of the executive function of cognitive flexibility and therefore concern the essential interpersonal ability of perspective taking (117). Since working memory is a precondition of “making sense of written or spoken language whether it is a sentence, a paragraph or longer” [(152), p. 143], deficits of ALS-patients can be assumed to make communication difficult. This is especially true for communication slowed down by HT-AAC-use and for such dealing complex issues. The latter also applies to an impaired ability of (abstract) reasoning which is reported to be common in ALS-patients (65, 125) and a cause of severe language comprehension deficits (126). All these deficits can be suspected to interfere with the ability to judge, which Flaherty-Craig et al. (125) directly assessed through an established cognitive battery and found to be impaired in a clinical relevant extent in 35% of the non-bulbar and over 50% of bulbar-onset-ALS-patients.

Taken together, executive dysfunction common in ALS-patients can be presumed to limit or rule out a clear, stringent, reliable, valid, effective, empathic or purposeful communication that is satisfying for both the patient and interlocutor, even when the patient is cognitively able to operate the AAC device. This high impact is supported by the negative association between subjective executive dysfunction and wellbeing of ALS-patients' caregivers (127).

### Social cognition deficits

Some of these aspects of successful communication should be importantly influenced by social cognition function as well. This domain includes the abilities to perceive, identify and understand, interpret or attribute social situations and other's cognitive and emotional states and to choose on that basis an appropriate reaction (67, 128, 129); abilities with an obvious importance for successful communication and social interaction and integration Deficits in this domain affect patients with ALSbi and ALSci (67, 99, 130), are associated with executive dysfunctions (67) but also occur in ALS-patients without those (130, 131). The results of a recent meta-analysis even suggests social cognition to be stronger compromised than executive functions (54).

Emotion recognition and Theory of Mind are most frequently studied in ALS-patients (67). Meta analyses report moderate deficits in facial emotion recognition for anger, sadness and disgust (132) and for disgust and surprise, respectively (67)–an inconsistency that can probably be explained amongst others reasons by the heterogeneity of the used measures and of the clinical and cognitive features of the mainly small study samples. A recent study confirmed deficits of correct emotion recognition in face as well as in voice even for ALS-patients with otherwise unimpaired cognitive abilities, but particularly for complex emotion expressions (133). Irrespective of the specific (negative) emotions though, a lacking ability of identifying and consequently attributing them correctly and responding to them adequately can be considered to be very dissatisfying for patient and interlocutor, causing frustration and interpersonal conflicts; all aspects possibly affecting HT-AAC use and validity of QoL measures of locked-in patients which has not yet been studied.

This is just as true for deficits in Theory of mind, a complex concept that includes the ability of perspective-taking (ToM-PT) according to understand other persons' behavior by representing their emotions and cognitions, e.g., thoughts and beliefs (134, 135). In accordance with findings for cognitive flexibility reported above, meta-analyses proved a lower performance of ALS-patients in different measures of ToM-PT compared to healthy controls (67, 132). This finding is confirmed by a recent study for early-stage ALS-patients (136) while again nothing is known yet in very advanced stages. Deficits are repeatedly reported to be more pronounced in ALS with bulbar onset (125, 132, 136, 137) and therefore in the subgroup of patients that is more frequently or earlier dependent of HT-AAC support for communication.

Considering that human behavior is crucially motivated and determined by emotional and social goals (128), the quantity, subjective quality and thereby value of communication can be assumed to suffer under discussed deficits. This is true for the ALS-patients themselves but especially for their next of kin, as the deficits potentially compromise the relationship, intimacy and their wellbeing and quality of life; like it is known to result from ALS-caused changes in behavior, cognition and communication in general (138).

This assumption is importantly supported by findings of changes in social behavior observed by primary caregivers: 70% showed an increased self-centeredness and a reduced interest for the feelings of others persons (139). A study by Fisher et al. (66) further suggests a lack of patients' insight into their social cognition and consequently social behavior impairment and therefore a lack of awareness of its effect on communication and interaction partners, which can be assumed to even increase the burden due to this impairment for the next of kin.

Additionally, the negative impacts of social cognition deficits can be presumed to be strengthened by general characteristics of the disease and of communication via HT-AAC: mimic and gestures are strictly limited, eye contact is not possible while speaking or to say writing, communication is slowed down immensely and the voice output does not transport any emotions.

### Memory impairment

Memory functions have been studied very frequently in ALS and deficits were found by a lot, although not by all studies (99). Focusing their importance for communication ability, immediate (54, 63) and delayed verbal memory are often severely impaired in ALS, also again when controlled for reduced motor speed (54). Recent findings suggest that such deficits are independent from executive dysfunctions (140). Immediate and delayed prose memory (saying recall of stories) as a special type of verbal memory was found to be affected in over 20% of high-functioning ALS-patients (141). In accordance with word-finding and naming-deficits, disturbances in sematic memory seem to affect more than the half of the ALS-population (142).

### Behavioral changes

Despite cognitive deficits–although not independent from them and often hard to distinguish (108)–frontal lobe dysfunction is associated with various significant behavioral changes and neuropsychiatric symptoms in ALS, frequently disinhibition, mood disturbances, and in particular apathy (104, 108, 133, 139, 143–147). Regarding the issue of patient's motivation to communicate, studies by Lillo et al. (124, 143) for example found significant symptoms of apathy in ALS, particularly a crucially limited motivation in 80% and a significant apathy in about 40% of the 92 enrolled patients (143). These syndromes were reported by caregivers in the questionnaire CBI-R (148), which assesses motivation mainly according to social motivation, e.g., as the motivation to stay in contact with significant others, show affection to them and be interested in their issues and concerns. Therefore, this finding is in accordance and probably directly connected with deficits of social cognition and behavior illustrated before. For other measures, caregivers report a clinical relevant apathy for up 40–60% of the ALS-patients (139, 146, 147). The significance of apathy for communication and social interaction is in accordance with the finding that caregivers and next of kin report a reduced initiation of conversations by the ALS-patients compared to premorbid behavior (66, 146) to show a reduced initiation of conversations. It can be moreover assumed that a lack of motivation up to apathy might especially affect communication by means of HT-AAC, regarding the high effort that is required for training and use of such devices for communication purposes, e.g., choosing every letter of a message via eye movement. Not surprisingly, apathy is strongly associated with caregiver burden (133).

Depressive symptomatology is another factor that must be considered to compromise patients' motivation to communicate. A clinical relevant severity is reported for 30–60% of the ALS-patients (45, 51, 149, 150). Equivalent to dysexecutive syndrome, behavioral changes in ALS are negatively correlated with caregivers' psychological wellbeing (147).

### Consequences of cognitive and behavioral dysfunctions for HT-AAC

In summary it can be argued that frequent cognitive and behavioral deficits and impairments in ALS have a negative effect on communication in general and in particular by means of low and high tech AAC. Therefore, they form a mayor challenge for adapting those devices to the individual patient with the aim of maintaining and supporting subjective value of and motivation for communication in both patients and communication partners. Changes in cognitive function should thus be monitored continuously, on the one hand to support this continuous adaption process and on the other hand to prepare patients and next of kins for upcoming challenges and (further) limitations of communication possibilities (91).

In the case of LIS, this objective is particularly challenging and at the same time very important to be achieved. Challenging because it requires motor and speech free tests and thus emphasizes the significance of developing eyetracking-based neuropsychological tests. Important, first because a restriction of direct communication via HT-AAC due to cognitive or behavioral deficits cannot or hardly be compensated by indirect communication in form of, for example, gestures and mimic. Second, because tests suitable for LIS-patients are needed to understand the natural history of ALS; referring to the Braak staging system in particular (151–153), this means to understand whether the progressive pathological involvement of brain structures, including such responsible for cognitive functions especially in late ALS-stages, continues also in the stage of LIS until TLIS. Third, because of the relevance of cognitive impairment for the highly important conclusions from discrepancies between patient's and next of kin's opinion concerning QoL and life prolonging measures. Concerning possible adaptions of HT-AAC devices for communication purposes to cognitive limitations, language dysfunction is–at least for mild to moderate severity–most likely the easiest part to compensate by high-tech devices. (Individualized) word prediction and word and sentence templates can facilitate language production and comprehension. A possible adaption of HT-AAC devices e.g., for LIS-patients with aphasia is the use of a symbol-based interface, which allows patients to express at least basic needs and wants and to control technical devices like TV, radio or lights.

However, deterioration of cognition can make the use of HT-AAC impractical (18), in particular when patients progress into a FTD. It can be additionally assumed that frontal dysfunctions adversely interfere with the patient's motivation as well as the ability to judge the need for using HT-AAC devices for communication, based on a lack of insight e.g., in the non-comprehensibility of the own spoken language. This idea is indirectly supported by data suggesting an association between cognitive and behavioral impairment and low compliance with treatment in ALS (145).

Impaired cognitive and especially high cognitive functions like reasoning and social cognition that might crucially limit the ability to judge play moreover an especially critical or even devastating role when it comes to decisions over life-prolonging measures (108), concerning reliability and validity of such decision in view of the discussed importance of HT-AAC for making them autonomously. This is particularly true when patients' and next of kin's opinions in this matter diverge, considering the consequences of such decisions also for the family and the patient's beloved ones.

The authors believe that it is therefore highly important to clearly diagnose cognitive and behavioral disturbances also in advanced disease stages including LIS. Having a clear diagnosis of dementia or cognitive or social impairment enables the responsible care takers or medical doctors to draw the right conclusions. On the side of the caretaker, this can mean to correctly interpret the patient's unsatisfying (e.g., diminished or non-empathic) communicative behavior, this is to say as a consequence of the disease, which can be relieving. Discussed findings of rejection of ETCS devices by family members and their higher burden after the patient's supply with the communication device (37, 44) support this idea. On side of the caretaker and the attending physicians, drawing the right conclusions might also mean to decide to limit life-prolonging measures. Concerning such decisions with regard to the patient's will, cognitive diagnostic and an earliest possible psychoeducation for patients and next of kin/caregiver about the frequent cognitive and behavioral deficits of ALS is important: first, to emphasize the need to continuously clarify and record this will in written (e.g., in a patient decree), since cognitive impairment might inhibit a reliable or valid decision at some points; second to allow patients and their families to take in account possible severe cognitive decline in future as an explicit factor for such life-prolonging/ending decisions (e.g., the will to end life prolonging measures in case of FTD or when the patient is not able to communicate via HT-AAC anymore).

## Brain-computer interfaces

Discussed limitations and shortfalls of ECTS systems as means for communication and environment control raise the question whether there are alternative technological HT-AAC approaches. Brain computer interfaces (BCI) could in theory be one answer, particularly for the mentioned subgroup of (long-surviving) ALS-patients in whom the usability of ETCS is compromised by oculomotor dysfunctions (76), gaze fatigue (154) or the loss of eye movement control in TLIS (155). BCI systems enable e.g., computer operation by voluntary modulation of one's own brain activity which is decoded into commands (e.g., selection of an item) without requiring any motor control (156–158). They are therefore considered a promising communication tool for advanced ALS or LIS-patients, respectively (159–162) and the only remaining option for TLIS-patients (163) or those with severe gaze dysfunction in general (82). It is another advantage over ETCS systems that BCI systems don't require still and strict frontal positioning to the screen (164).

While invasive BCI methods like intracortical electrodes have been primarily studied in animal research (165) and infrequently in tetraplegic patients (166), a number of non-invasive BCI systems has been evaluated in severely paralyzed patients including ALS-patients in (T)LIS (98, 160, 162). The majority of these systems have been developed for spelling or writing or texting (167) which is allowed by selection of letters, words or phrases presented on a screen (98).

In this context, reviews valuate non-invasive BCI based on EEG as a practicable, promising and the most widely used approach (98, 168). Also ALS-studies provide evidence for the principle feasibility of such systems for a relevant proportion of patients. Those BCI devices are based on shifting of particular brain responses measured as EEG-parameters: slow cortical potentials (169–171), sensimotor rhythms (SMR) (155, 172, 173) and the event related potential P3 (164, 173–181).

Communication is one of the BCI-functions that ALS-patients are mostly interested in (182) and with the focus of this review on the importance of communication in palliative care and thus on spelling BCI systems, P3 is the most frequently used and studied EEG parameter (98). The principle of most (P3-)BCI-spelling protocols is the following (98): an e.g., 6 × 6 matrix of items, usually letters, is presented on a screen and the patient is instructed to concentrate on the target item. Different rows or columns flash rapidly in succession. The P3 can be measured about 300 ms after the item flashes and by averaging the P3-amplitudes following each flash, the target item can be identified [e.g., (173, 175)].

Usual objective evaluation criteria for such BCI are the effectiveness, i.e., classification accuracy (CA) defined as the “percentage of correct target selection” (183) and the efficiency (spelling speed). People with ALS declare a CA-threshold of 90% as satisfying (184). On this basis, all of the 20 ALS-patients in a recent study by Guy et al. (164) achieve a satisfying CA in the simpler task of copying a text (“copy spelling”), although it was lower that 90% for writing a text of their choice (“free spelling”). Anyway, patients reported an overall high user satisfaction (average 8.7 on a 10-point-scale). However and importantly, dysarthria was no inclusion criteria for the study, no subject was defined as (T)LIS and all showed unimpaired gaze control. This is in accordance with a mean CA of 92% reported by Pires et al. (179) for a classical spelling paradigm, whereby they included almost exclusively early-stage ALS-patients with even lower physical disability. A study of more severely motor impaired but also visually unimpaired ALS-patients (*N* = 14) only reported the maximum accuracy: it was circa 96% and did not differ significantly between patients and age-matched controls (175). In a previous study conducted by the same research group, 17 of the 25 enrolled patients achieved a high accuracy (average CA 92%), but an accuracy below 40% for the remaining 8 patients indicates no usability of the BCI for communication; importantly, the latter patients all suffered from some type of visual dysfunction (176).

Overall, however, most studies report for ALS-patients with varying disability-levels and without controlling for visual deficits accuracy-rates that fall significantly below the 90% threshold (155, 170, 172–174, 181, 185). This is in line with the average CA of 73.7 %, reported in a meta-analysis by Marchett and Priftis (183). Although higher spelling accuracy for able-bodied/healthy controls than for patients is reported (186), no evidence for a worse performance in ALS-patients with higher compared to those with lower physical disability is provided by very few studies with a sufficient sample size for analyzing this influence (172, 175, 176). For (T)LIS-patients in particular though, there are only few and only case studies; two of them actually found high and stable effectivity of and satisfaction with a P3-BCI-system for spelling (180) and painting (187), while one reports several unsuccessful trials of implementing a BCI in one patient transferring from LIS to TLIS (163).

An efficacy-related problem that would crucially compromise the BCI-usability in the context of palliative care for patients with such a quickly progressing disease like ALS are the very long training sessions required for reaching outlined accuracies (179). Another practical issue would be the long time that is needed to set up an EEG-BCI (164).

With respect to efficacy in potential future everyday use of BCI arises another main problem: the consistently reported low efficiency of spelling, i.e., in real-life use the slow potential communication speed. While ALS-patients indicate a spelling rate of 15–19 words per minute as satisfying (184)–with a word is standardized to consist of five letters on average (188)–rates in recent P3-BCI-studies range between 2.1 words and 5.0 words (164, 175); and are even much lower (one letter, i.e., about 0.2 words per minute) for EEG-systems using SCR (171) and SMR-modulation (189). This problem is qualified by the patients mostly high satisfaction with BCI though (164, 171)–a finding which supports that speed is less relevant for (T)LIS-patients than the possibility to communicate at all and in a reliable manner (180, 184).

With regard to obligatory decision in palliative care and particularly those regarding life-ending-choices, even a BCI for yes/no-questions could be crucial for these patients without any other possibility to express their needs and decisions–but a very high validity and reliability would be even more essential for this purpose Chaudhary et al. (162) were the first to evaluate a BCI for yes/no-answers in 4 TLIS- or patients transferring from LIS to TLIS, which relies on measuring change in frontocentral hemoglobin. The correct-response rate about 70% is still very unsatisfying although it could be valued as a promising base for further developments. In conclusion of this chapter it is important to note that a lot of the described pitfalls (see chapter Limitations and pitfalls of HT-AAC-use and -supply) and especially those due to cognitive impairment (see chapter Cognitive and behavioral impairment and its consequences for HT-AAC-use) account for BCI use as well.

## Future directions

From the reviewed literature it can be concluded that there is tremendous need for further research on the impact of HT-AAC, technical progress of the devices and for an increased awareness of upcoming opportunities and the importance of communication on wellbeing by professionals caring for severely disabled patients and by policy-makers.

### Future technological developments

A main obstacle for mobile use of ETCS is that they are bound to be used in conjunction with a computer screen. Eyetracking devices are typically mounted at the bottom of the computer screen, on which the user interface, e.g., an on screen keyboard, is displayed. In the near future with advances in augmented and virtual reality, head mounted systems with built in eyetracking capabilities may be applied. In addition to the advantage of being more portable, a see-through display would have the benefit of allowing the user to look at its communication partner and vice versa during conversation. In nowadays systems the computer screen is blocking the line of sight between the two partners, leading to subjectively reduced closeness as described above [e.g., (190)]. In order to reduce the sensitivity of ETCS to adverse lightning conditions, non-infrared based camera approaches may be used in the future, although they have not yet provided the level of accuracy that is needed for good gaze control.

We conclude from the discussed reports that existing (P3-)BCI systems for spelling/communication purposes do not allow and are not suggested for use in standard palliative care of ALS-patients at this point of time, especially in light of patient's quite high expectations on BCI-use (182). Concerning on the one hand patients that are (still) able to use ETCS, this conclusion supports the statement of Marchetti and Priftis (183) that (P3-based) BCIs for spelling still have many disadvantages and no clear advantage that would feature them as an alternative communication tool in daily use. It is however important to note already existing modifications of visual stimuli presentation (174, 179, 185) and technical improvements for existing BCIs (177) that increase their accuracy significantly. Kaufmann and colleagues (185) for example could increase brain responses and consequently CA by integrating well known faces in the matrix in addition to the letters.

Concerning on the other hand ALS-patients that can't use ETCS anymore, BCI systems need to be primarily more effective and secondarily more efficient than they are at the moment, but would be then highly significant for this patient subgroup. Moreover, concerning TLIS-patients as well as the evidence for lower spelling accuracy because of visual problems, there is an indication for non-visual BCIs. Auditory or tactile BCIs exist, but are less widely studied up to now (191, 192). A case study of a LIS-patients found clear superiority of tactile modality (185), while a comparison between a visual and equivalent auditory P3-system indicates the latter as a still less accurate but still promising option for LIS- and TLIS-patients with visual deficits (155). In accordance with that, a LIS-patient with subjectively worsening gaze control expressed in a case study of Käthner et al. (82) his preference for an auditory BCI over ETCS, although the latter showed significantly higher accuracy rates and communication speed.

BCI are therefore an important field of research with regard to the objective to secure self-determination and QoL in every, including the terminal phase of life of patients with most severe disabilities. So far, very few case studies explored BCI usability outside an experimental setting (187, 193). One of these studies though even found evidence for a relevant positive impact of BCI-use for spelling on QoL of a single TLIS-patient (193). Future studies need to examine larger and more samples of (T)LIS-patients in their living environment and everyday life.

The development of inexpensive hard- and software that can be easily adapted to multiple access modes and customized to the patients' individual needs should be a general goal. In the COGAIN (“communication by gaze interaction”; www.COGAIN.org) European Network of Excellence professionals and researchers collaborate toward developing advanced gaze based communication technologies in order to enhance applicability and user satisfaction of the devices and ensure quality control in patient care and research (194).

### Health policy and attitudes

In addition to ALS and other motor neuron diseases, there is a high potential for HT-AAC to improve care for patients with other acquired neurological conditions that lead to impaired communication abilities, e.g., traumatic brain injury, brainstem impairment, severe chronic aphasia and apraxia of speech, primary progressive aphasia, and dementia (18). Depending on the particular type and extent of communication and/or motor and/or cognitive impairment that are caused by these conditions, different kinds of AAC-systems and functions can be assumed the most useful ones for the patient (e.g., typing vs. eyetracking communication devices; auto-correction function for aphasia patients). Based on an epidemiological approach, Creer et al. (195) estimated the prevalence of people who could benefit from AAC technologies in the UK at 0.5%.

Enabling the individual's optimal communication capabilities should be the standard of care in order to maintain QoL and self-determination in the comprehensive and palliative care for all human beings including severely disabled patients. The German treatment guidelines for ALS (https://www.dgn.org/leitlinien/3012-ll-18-ll-amyotrophe-lateralsklerose-motoneuronerkrankungen) contain the general information that in case of dysarthria, dynamic AAC technologies with speech output and environment control should be procured. However, the guidelines do not offer detailed recommendations for assessment of communicative abilities for AAC evaluation and supply and do not refer to their value for patients' QoL. Moreover, they are not legally binding.

Furthermore, advance care planning in ALS should explicitly consider the possibility that patients' can reach a disease state in which communication is not possible at all. Advance care planning and power of attorney for caregivers, also including the termination of life-sustaining measures in ALS and other severe neurological disorders, is however a complex issue and thus beyond the focus of this review.

## Conclusions

Usually, the term “palliative care” is not associated with high-technologies, probably because they are supposed to contribute to the dehumanization of medicine and the superiority of survival over QoL. However, HT-AAC devices are not conceived to prolong survival, but to enhance QoL and autonomy for the remaining lifetime which is a core component of palliative care. These HT-AAC devices thus should play an exceptional role in palliative care compared to many other high-tech devices normally used to prolong survival.

HT-AAC have a high potential for improving palliative care for people with ALS and other severe diseases that lead to impaired communication abilities. Several studies convincingly demonstrated that complex and caregiver-independent communication is enabled by HT-AAC, which is crucial for addressing psychological, spiritual, and essential issues in palliative care. Within the current knowledge, the use of HT-AAC respectively the optimization of patients' ability to communicate leads to improved QoL and better wellbeing and enables the maintenance of social roles and intellectual stimulation. Moreover, communication is essential for the prevention of patient autonomy concerning end-of-life care and decisions. The use of HT-AAC can therefore lead additionally to reduced caregiver-burden and strengthen family cohesion, which however needs further independent investigations, also concerning critical issues like barriers of acceptance of the devices.

The technology does also still possess unresolved pitfalls. These can be grouped by different aspects:

- Technically, limitations mainly arise from the infrared camera system with respect to distinct light conditions (mainly outside), wearing of glasses and body positioning.
- Disease conditions such as cognitive, e.g. executive or social cognition deficits up to advanced dementia, language impairments including aphasia, but also TLIS or other eye-gaze alterations obviously raise difficulties.
- Critical issues which can be solved more easily are such as barriers of acceptance amongst patients and caregivers, lack of awareness by both health care professionals and politicians/social system and the lack of clear and binding guidelines. The latter is also important to oblige HT-AAC providers to continually support the customer.

Healthcare professionals, technology providers as well as policy makers need a greater awareness of the possibilities but also of possible pitfalls of HT-AAC technologies. They are required to enable timely access to adequate, user-friendly and individually tailored equipment and provide ongoing training, customization and support (14), without letting quality of support suffer at the expense of cost effectiveness. This can be best achieved by individual evaluation of the patients' needs and concerns and by sufficient and continuous training in handling of the devices. It also includes the retraction of HT-AAC devices under certain circumstances, which might be severe dementia, development of significant gaze palsy or TLIS or also the patient's wish to return the device, which should optimally be properly assessed by means of the HT-AAC device.

On the basis of past and future research, detailed and binding guidelines that support patients' supply with AAC devices should be developed in order to ensure effective communication. Patients have to be enabled to make informed decisions for or against any communication support in order to allow the longest period of lifetime with the best possible QoL in accordance with their free will and their individual aims and wishes.

There is tremendous need for further research on the impact of HT-AAC, technical progress of the devices and for an increased awareness of upcoming opportunities and the importance of communication on wellbeing by professionals caring for severely disabled patients and by policy-makers. The consideration of HT-AAC interventions should be embedded as mandatory in multidisciplinary palliative care in order to enable autonomy by ensuring access to the best individually tailored communication strategies and their adjustment to changing needs of patients with ALS.

## Author contributions

All authors listed have made a substantial, direct and intellectual contribution to the work, and approved it for publication.

### Conflict of interest statement

KL, EA, and AH have nothing to report. MJ's affiliation “Interactive Minds Dresden GmbH” is a provider of ETCS in the region of Dresden.

## Abbreviations

(HT-)AAC: (high-tech) augmentative and alternative communication
ALS: Amyotrophic lateral sclerosis
BCI: brain-computer-interfaces
CA: classification accuracy
ETCS: eye-tracking computer systems
FTD: frontotemporal dementia
(T)LIS: (total) locked-in-state
NIV: non-invasive ventilation
PEG: percutaneous endoscopic gastrostomy
QoL: quality of life
TIV: tracheostomy invasive ventilation.
